# Δ9-tetrahydrocannabinol exposure during rat pregnancy leads to symmetrical fetal growth restriction and labyrinth-specific vascular defects in the placenta

**DOI:** 10.1038/s41598-019-57318-6

**Published:** 2020-01-17

**Authors:** Bryony V. Natale, Katarina N. Gustin, Kendrick Lee, Alison C. Holloway, Steven R. Laviolette, David R. C. Natale, Daniel B. Hardy

**Affiliations:** 1 Department of Obstetrics and Gynaecology, Queen’s University, Kingston, Canada; 2 Department of Obstetrics, Gynecology & Reproductive Sciences, School of Medicine, University of California San Diego, La Jolla, CA USA; 3 Department of Biomedical and Molecular Sciences, Queen’s University, Kingston, Canada; 4 Department of Obstetrics and Gynecology, McMaster University, Hamilton, Canada; 5 Department of Anatomy and Cell Biology, London, Ontario N6A 5C1 Canada; 6 The Children’s Health Research Institute, London, Ontario N6A 5C1 Canada; 7 Lawson Health Research Institute, London, Ontario N6A 5C1 Canada; 8 The Departments of Obstetrics and Gynaecology, London, Ontario N6A 5C1 Canada; 9 The Departments of Physiology and Pharmacology, London, Ontario N6A 5C1 Canada; 10 The University of Western Ontario, London, Ontario N6A 5C1 Canada

**Keywords:** Intrauterine growth, Outcomes research

## Abstract

1 in 5 women report *cannabis* use during pregnancy, with nausea cited as their primary motivation. Studies show that (-)-△9–tetrahydrocannabinol **(Δ9-THC**), the major psychoactive ingredient in *cannabis*, causes fetal growth restriction, though the mechanisms are not well understood. Given the critical role of the placenta to transfer oxygen and nutrients from mother, to the fetus, any compromise in the development of fetal-placental circulation significantly affects maternal-fetal exchange and thereby, fetal growth. The goal of this study was to examine, in rats, the impact of maternal Δ9-THC exposure on fetal development, neonatal outcomes, and placental development. Dams received a daily intraperitoneal injection (**i.p**.) of vehicle control or Δ9-THC (3 mg/kg) from embryonic (**E**)6.5 through 22. Dams were allowed to deliver normally to measure pregnancy and neonatal outcomes, with a subset sacrificed at E19.5 for placenta assessment via immunohistochemistry and qPCR. Gestational Δ9-THC exposure resulted in pups born with symmetrical fetal growth restriction, with catch up growth by post-natal day (**PND**)21. During pregnancy there were no changes to maternal food intake, maternal weight gain, litter size, or gestational length. E19.5 placentas from Δ9-THC-exposed pregnancies exhibited a phenotype characterized by increased labyrinth area, reduced *Epcam* expression (marker of labyrinth trophoblast progenitors), altered maternal blood space, decreased fetal capillary area and an increased recruitment of pericytes with greater collagen deposition, when compared to vehicle controls. Further, at E19.5 labyrinth trophoblast had reduced glucose transporter 1 (**GLUT1**) and glucocorticoid receptor (**GR**) expression in response to Δ9-THC exposure. In conclusion, maternal exposure to Δ9-THC effectively compromised fetal growth, which may be a result of the adversely affected labyrinth zone development. These findings implicate GLUT1 as a Δ9-THC target and provide a potential mechanism for the fetal growth restriction observed in women who use *cannabis* during pregnancy.

## Introduction

Over the last decade, *cannabis* use has progressively increased in pregnant women, in part due to the perception that its usage poses no risk in perinatal life^1,2^. In the United States, the rates of self-reported or screened *cannabis* use in pregnant mothers (18–24 years) varies from 6 to 22%, with some women admitting to daily use^2,3^. Of great concern is that *cannabis* use in pregnancy is more prevalent in young, urban, socially disadvantaged women^4,5^. Three systematic reviews and meta-analyses have validated the relationship between maternal *cannabis* use and both low-birth weight and adverse neurodevelopmental outcomes^6–10^. These studies, however, are confounded by sociodemographic factors and that *cannabis* is often accompanied by use of other drugs^6–10^. To address the intrinsic limitations of those clinical studies, animal experiments have demonstrated that exposure of pregnant rodent dams to Δ9-THC, the major psychoactive component of *cannabis*, leads to placental dysfunction and low birth weight offspring^11,12^. This is alarming as the concentration of Δ9-THC in *cannabis* has steadily increased (from 3 to 22%) over the last two decades, and animal studies indicate that Δ9-THC crosses the placenta with 10–28% of maternal concentrations detected in the fetal plasma, and 2–5 times higher concentrations in fetal tissues^13,14^.

To date, the underlying molecular mechanisms for Δ9-THC-induced placental insufficiency are not completely understood. The molecular targets of action for Δ9-THC in the placenta are the two G-coupled cannabinoid receptors, CB1R and CB2R, which are part of the endocannabinoid system that plays a role in fertilization, embryo implantation, and early placentation^15,16^. In mouse, intraperitoneal injection (***i.p***.) of 3–5 mg/kg Δ9-THC both cause reduced fetal birthweight^12,17–19^. At 5 mg/kg, fetal demise^12^ was reported, with altered placenta development further described^12,18^. Specifically, placentae from exposed dams had an overall reduction in CB1R and CB2R expression in association with impaired placental angiogenesis, narrowing of maternal sinusoids and increased trophoblastic septa diameter in the labyrinth zone, while the junctional zone exhibited disordered spongiotrophoblast and fewer glycogen cells^12,18^. Conversely, the 3 mg/kg Δ9-THC dose did not lead to alterations in maternal behavior or physical measures^17,19^ and yielded Δ9-THC serum concentrations (8.6–12.4 ng/ml Δ9-THC) that are at the lower end of the range of that reported (i) in *cannabis* smokers (13–63 ng/ml from a 7% Δ9-THC content cigarette) 0–22 hours post inhalation, and (ii) in aborted fetal tissues (4–287 ng/ml) from pregnant *cannabis* smokers^20–22^.

Fetal growth restriction can result from impaired placenta development^23–25^ and the association between intrauterine growth restriction (**IUGR**) and the subsequent development of type 2 diabetes, obesity and metabolic syndrome (**MetS**) is often referred to as the “fetal origins hypothesis”^26–29^. Compromised nutrition and metabolism, in development, induce adaptations suited for survival short-term, but can become maladaptive if there is a ‘mismatch’ to the predictive postnatal environment, leading to long-term metabolic disease in adulthood^30^. Clinical reports suggest that after fetal growth restriction, there is often a period of post-natal catch-up growth, which significantly increases the risk of metabolic disorders^31–34^. The pregnant rat is an excellent model in which to study fetal growth restriction, reciprocating both post-natal catch-up growth and the onset of MetS^35–38^. As such, the aim of the current rat study was to use a dose of Δ9-THC that reports serum Δ9-THC concentrations that are within range of *cannabis* smokers^20–22^ with no reported fetal demise in order to investigate whether maternal exposure would lead to fetal growth restriction and post-natal catch-up growth. Given that maternal nicotine exposure during gestation results in fetal growth restriction associated with placental insufficiency^25^, we sought to investigate whether structural or vascular defects in the placenta might also be occurring. Moreover, as fetal growth restriction can occur via impaired transport of key nutrients to the fetus^39–45^, we further characterized the effects of Δ9-THC on the expression of the placental glucose transporter (**GLUT1**) and its upstream regulator, the glucocorticoid receptor (**GR**).

## Results

### Δ9-THC exposure in the rat does not affect maternal weight or food intake

Pregnant rat dams received either daily doses of vehicle or Δ9-THC (3 mg/kg *i.p*.) from embryonic day 6.5 (E6.5) through E22. To evaluate maternal outcomes, gestational length, average food intake, pregnancy weight gain, litter size and live birth index were measured. In agreement with previous rodent studies^12,19,46,47^, daily administration of Δ9-THC to pregnant dams had no effect on maternal weight gain during pregnancy, or maternal food intake (Table 1). In addition, Δ9-THC (3 mg/kg *i.p.)* did not alter gestational length, litter size or live birth index similar to previous studies with maternal Δ9-THC exposure (Table 1)^46,47^.

**Table 1:** Maternal and neonatal outcome measurements.

Maternal/Neonatal Outcome Measures	Vehicle	Δ9-THC	p-value
Gestational Length (days)	21.6 ± 0.33	22	0.37
Average Food Intake: days 12–14 (g/day)	22.9 ± 0.7	19.1 ± 2	0.28
Average Food Intake: days 18–20 (g/day)	30.3 ± 1	27.4 ± 0.1	0.06
Pregnancy Weight Gain: GD6-GD22 (g)	118.2 ± 20	103.9 ± 11	0.56
Litter Size (n)	13.3 ± 0.6	11 ± 0.3	0.14
Live Birth Index (%)	100	96.7 ± 3	0.37
Pup Weight (g)	7.01 ± 0.11	6.6 ± 0.1	**0.001**
Survival to PND4 (%)	100	100	1

### Maternal Δ9-THC exposure results in symmetrical IUGR

**Figure 1:**
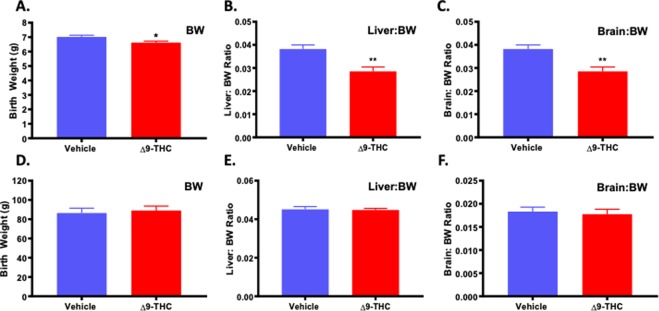
Exposure to 3 mg/kg Δ9-THC during gestation leads to symmetrical fetal growth restriction followed by postnatal catch-up growth. (**A**) birth weight, (**B**) liver:body weight ratio at birth, and (**C**) brain: body weight ratio at birth. (**D**) body weight at 3 weeks, (**E**) liver:body weight ratio at 3 weeks, and (**F**) brain: body weight ratio at 3 week. Mean ± SEM, average weight/litter, N = 8 dams/group, Significance; Student’s t-test (*P < 0.05, **P < 0.001).

To determine the effect of Δ9-THC exposure on neonatal outcome, assessments included pup weight, and organ to body weight ratio (hallmarks of growth restriction) along with survival to post-natal day (**PND**)4. A small for gestational age (**SGA**) birth is <10^th^ percentile for gestational age, or more than 2 standard deviations below the mean, while Intrauterine Growth Restriction (**IUGR**) refers to a reduction in expected fetal growth^48^, thus, not all IUGR births are SGA^48–51^. Further, growth restriction can be asymmetric, meaning there is first a restriction of weight, followed by length with a “head sparing” effect^51^. This is the most common form of IUGR, and is seen with pre-eclampsia, hypertension and uterine pathologies^51,52^. Symmetric growth restriction affects all growth parameters and affects the fetus in a uniform manner and can result in permanent neurological consequences. Symmetric growth restriction is more often the result of genetic causes, intrauterine infections and maternal alcohol use^51,52^. At birth, the pups from Δ9-THC exposed pregnancies were growth restricted and weighed significantly less than the vehicle control pups (p = 0.001; Table 1). Moreover, in the Δ9-THC group, 2 out 8 dams had one pup that was small for gestational age (SGA) (<2 STD of mean body weight) while the vehicle group had none. Building on a previous study that identified that exposure to *cannabis* smoke lead to impaired fetal organ development^53^, **PND1** neonates were sacrificed to examine organ-to-body weight ratios and Δ9-THC pups exhibited a ~25% decrease in both liver-to-bodyweight ratio and brain-to-bodyweight ratio (p < 0.01), indicating symmetrical IUGR (Fig. 1). However, the reduced fetal size of the pups from the Δ9-THC exposed pregnancies did not affect survival to PND4 (Table 1).

### Pups from Δ9-THC exposed pregnancies experience post-natal catch-up growth

As we have previously demonstrated that post-natal catch up growth in the rat exacerbates the incidence of MetS^54–56^, the pups were evaluated to see if they might be at increased risk. At PND21, pups from the Δ9-THC exposed pregnancies had exhibited catch-up growth with no significant difference in weight, liver to weight ratio or brain to weight ratio (Fig. 1).

### Placental weights increased at E19.5, with reduced fetal to placental weight ratio

To explore whether changes in placental structure and composition may underlie the fetal growth restriction observed, a cohort of vehicle and Δ9-THC exposed pregnant dams were sacrificed at E19.5 and fetal and placental weights were evaluated. Similar to PND1, the litter size at E19.5 was not altered between vehicle and Δ9-THC exposed dams (Table 2), nor was the number of reabsorptions significantly different (Table 2). The fetal weights in both treatment groups were the same, suggesting that the overall growth restriction identified at birth, takes place after E19.5. The fetal to placental weight ratio can be used as a measure of placental efficiency^57,58^. The placentae from Δ9-THC exposed pregnancies were significantly larger than the placentae from vehicle control exposed dams (p < 0.001), causing the fetal to placental weight ratio to be reduced (p < 0.05, Table 2).

**Table 2:** Fetal and placental outcome measurements at E19.5.

Fetal/Placental Outcome Measures	Vehicle	Δ9-THC	p-value
Litter Size	8.2 ± 1.7	8.8 ± 2.1	0.82
Number of Reabsorptions	0.25 ± 0.25	2.2 ± 1.03	0.11
Fetal Weights (g)	1.7 ± 0.11	1.9 ± 0.1	0.17
Placental Weight (g)	0.46 ± 0.02	0.58 ± 0.02	**0.0009**
Fetal:Placental Weight Ratio	3.66 ± 0.14	3.19 ± 0.12	**0.02**

### Structure and composition of trophoblast cells of the junctional zone were unaltered in placentae from Δ9-THC exposed pregnancies

**Figure 2:**
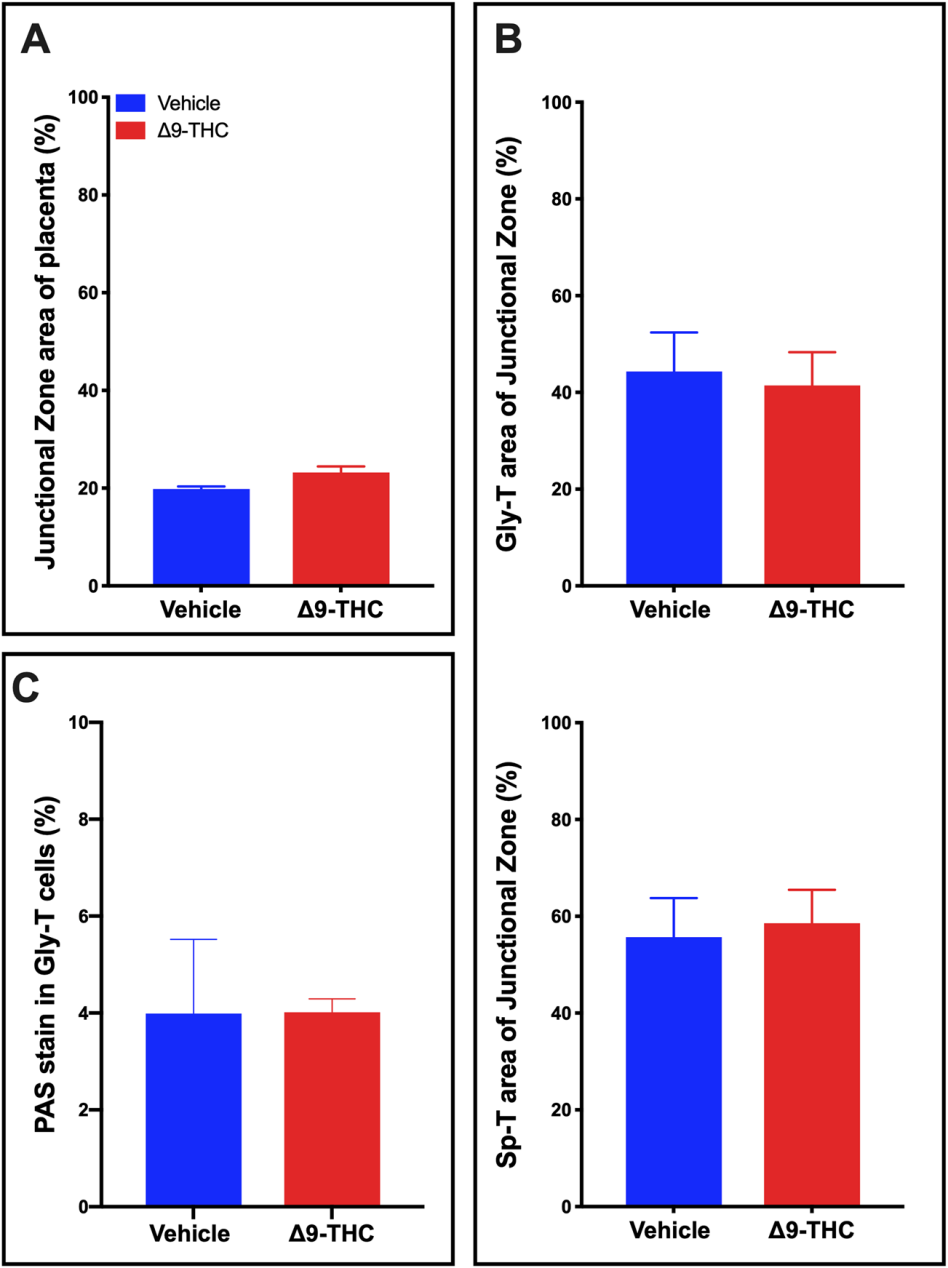
Exposure to 3 mg/kg Δ9-THC during gestation has no measurable effect on junctional zone size or composition at E19.5. (**A**) Percentage of junctional zone area of total placenta. (**B**) Analysis of the glycogen trophoblast (Gly-T) and spongiotrophoblast (Sp-T) complement of the junctional zone. (**C**) Percentage of PAS staining in Gly-T in junctional zone. For junctional zone, 6-images/placenta were taken at 10x. Graphs present mean ± SEM.

To determine whether structural changes in the placenta contributed to the increase in placental weights, histological assessment of the placental layers was performed. There was no change in the relative size of the junctional zone (Fig. 2A) between the vehicle treated controls and Δ9-THC exposed groups. Furthermore, histological analysis revealed no difference in the junctional zone composition of glycogen trophoblast (Gly-T) or spongiotrophoblast (Sp-T) populations (Fig. 2B**)** that make up this layer. It is worth noting that while 3 mg/kg Δ9-THC *i.p*. in the rat did not alter these populations, in mice, pregnancy exposure of 5 mg/kg Δ9-THC *i.p*. reported junctional zone disorganization with reduced glycogen trophoblast and the spongiotrophoblast populations^12^. It is possible that the higher dose of Δ9-THC may be more toxic to the junctional zone trophoblast and that 3 mg/kg allows for junctional zone specific trophoblast survival, though it must be considered that it could be a difference between species.

Given that Gly-T in the junctional zone store glycogen and storage can be altered in placentae that are functionally abnormal, we examined whether there was a greater accumulation of glycogen or aldohexoses in the placenta, as observed in other models of placental insufficiency^59^. PAS staining was performed on serial placental sections without and with diastase treatment to assess for the levels of total aldohexoses vs aldohexoses without glycogen, respectively. In the junctional zone of Δ9-THC placentae, diastase treatment confirmed that glycogen accumulated normally in glycogen trophoblast and that there was no difference in total aldohexoses between vehicle and treated placentae (Fig. 2C).

### Larger labyrinth layer, with a reduction in EPCAM+ labyrinth progenitors in placentae from Δ9-THC exposed dams

**Figure 3:**
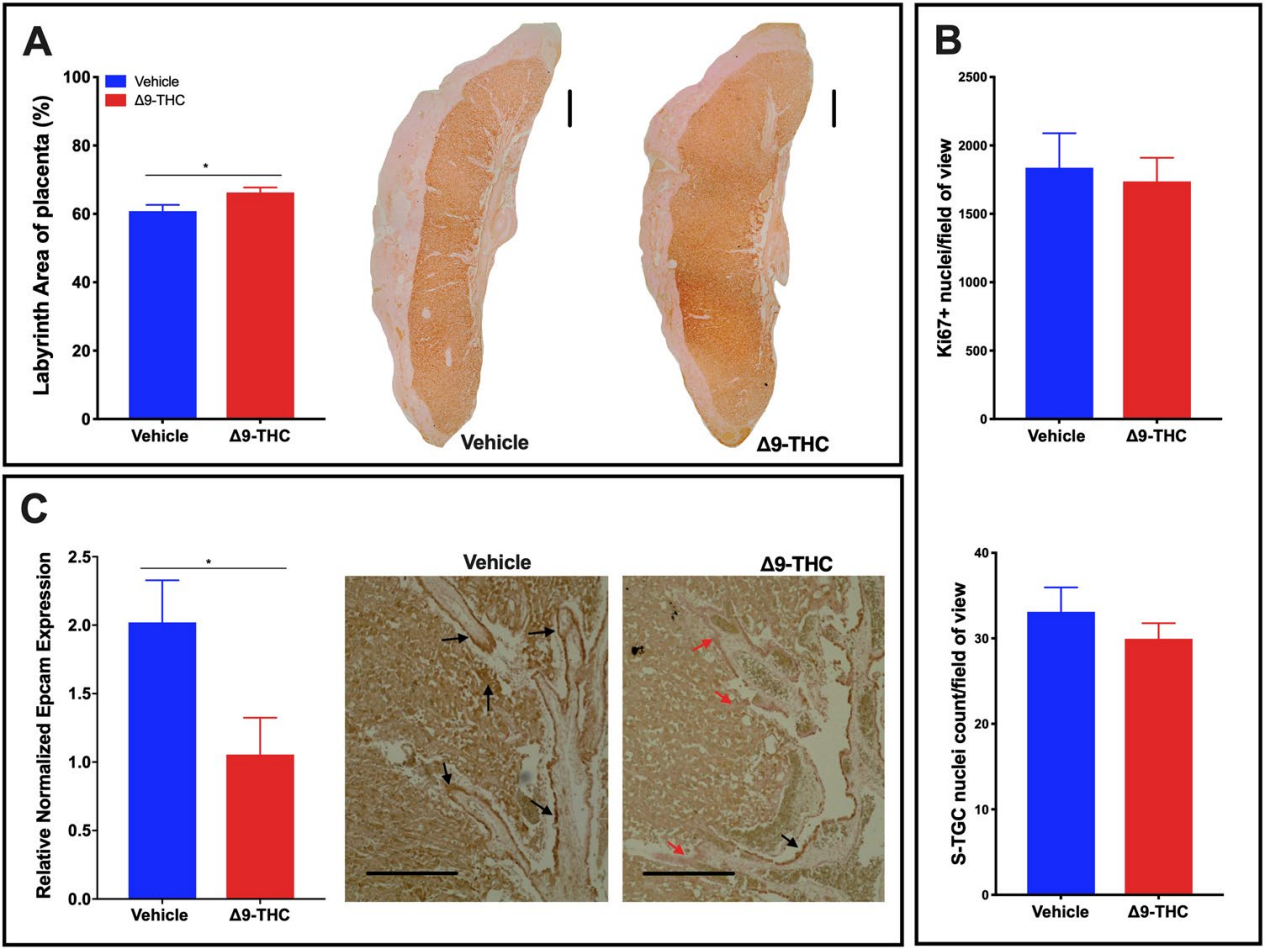
Exposure to 3 mg/kg Δ9-THC during gestation leads to increased labyrinth layer area at E19.5 compared to vehicle treatment, however with no associated increases in cell proliferation nor number of S-TGCs. (**A**) Percentage of labyrinth layer area of total placenta and representative images showing Iso-Lectin B4 staining in labyrinth. (**B**) Analysis of numbers of Ki67+ nuclei and S-TGC nuclei in the labyrinth layer. (**C**) Quantification of *Epcam* mRNA in rat placenta at E19.5 by qPCR (graph) and assessment of EPCAM protein expression by IHC in labyrinth layer. For labyrinth area, 6-images/placenta were taken at 10×, while for Ki67, S-TGC and Epcam assessment, 6-images/placenta were taken at 40×. Graphs present mean ± SEM. Significance; Student’s t-test (*P < 0.05). Scale bars = 500 uM in (**A**), 150 uM in (**C**).

Histological assessment of the placental layers revealed that the relative area of the labyrinth layer was increased in the placentae from Δ9-THC exposed dams (p < 0.05; Fig. 3A). It has been shown in the rat placenta that proliferation is highest in the labyrinth at E10-11 and has dropped to a basal level by E16^60^. At E19.5, proliferating cells are much less likely to be observed; however, as proliferation can be altered in response to placenta stress^61^, it was evaluated to see whether, the rate of proliferation, albeit low, was changed. The increased size was neither attributed to an increase in proliferation, as the number of Ki67^+^ nuclei was not altered (Fig. 3B), nor the number of sinusoidal trophoblast giant cells (S-TGCs) as there was no difference between treatment groups (Fig. 3B). Interestingly, the EPCAM^+^ trophoblast progenitor cells that give rise to the differentiated trophoblast of the labyrinth layer appeared fewer in the placentae exposed to Δ9-THC, and qPCR assessment, confirmed that *Epcam* expression was reduced (p = 0.04600) in response to exposure (Fig. 3C). To further investigate this finding and to determine if syncytiotrophoblast, which differentiate from EPCAM+ trophoblast precursors, were affected, we assessed the expression of *Gcm1* by qPCR. Interestingly, *Gcm1* expression was not altered by Δ9-THC exposure (Supplemental Fig. 1).

### Following gestational Δ9-THC exposure, rat placentae exhibit vascular defects

**Figure 4:**
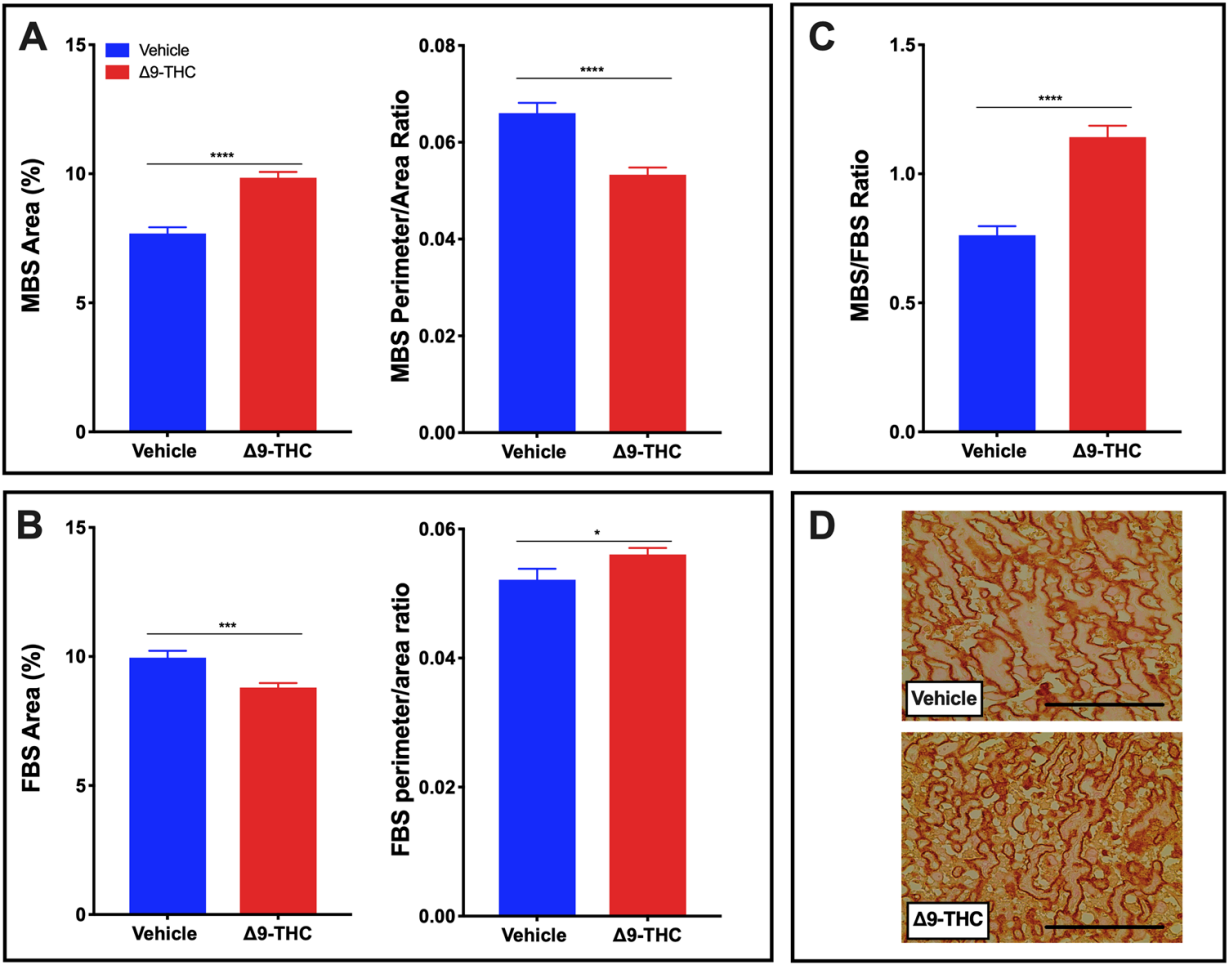
Exposure to 3 mg/kg Δ9-THC during gestation leads to increased maternal blood space to fetal blood space ratio in the labyrinth zone at E19.5 compared to vehicle treatment. (**A**) Percentage of maternal blood area and maternal blood space perimeter/area ratio in labyrinth zone. (**B**) Percentage of fetal blood area and fetal blood space perimeter/area ratio in labyrinth zone. (**C**) Maternal blood space to fetal blood space ratio in labyrinth zone. (**D**) Representative images of fetal blood spaces identified by Iso-Lectin B4 staining. 6-images/placenta were taken at 40×. Graphs present mean ± SEM. Significance; Student’s t-test (*P < 0.05, **P < 0.01, ***P < 0.001 ****P < 0.0001). Scale bar = 100 uM.

The labyrinth zone is the site of maternal-fetal exchange and alterations in vascular development are critical and can contribute to fetal growth restriction. To explore whether the fetal growth restriction observed in Δ9-THC pups could be attributed to placental insufficiency, the fetal capillary network and maternal blood sinusoids within the labyrinth layer (herein referred to as fetal and maternal blood spaces, respectively), were assessed^62–65^. The assessment included: area of blood spaces as a percentage of the field of view; maternal to fetal blood space ratio and the perimeter to area ratio, all indicators of surface available for nutrient exchange. The maternal blood space area was increased (p < 0.0001) in response to Δ9-THC exposure, with the perimeter/area ratio of the maternal blood spaces reduced (p < 0.05; Fig. 4A). Furthermore, the fetal blood space area was reduced (p < 0.001) in the placentae from Δ9-THC exposed dams, with an increased fetal perimeter to area ratio (p < 0.05; Fig. 4B,D). Collectively, the maternal/fetal blood space ratio was increased in the labyrinth zone of Δ9-THC placentae (p < 0.0001; Fig. 4C).

**Figure 5:**
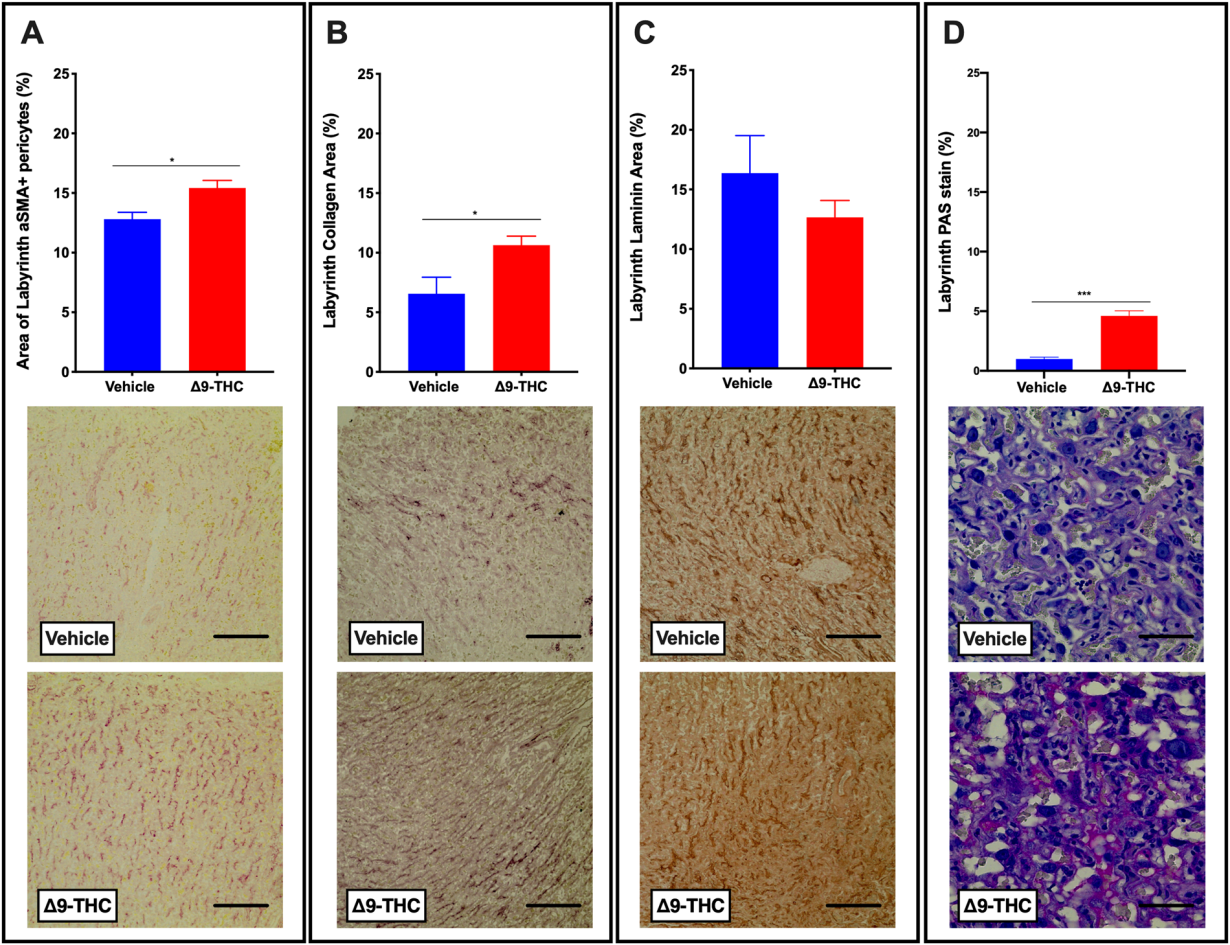
Exposure to 3 mg/kg Δ9-THC during gestation leads to increased pericyte and collagen area in the labyrinth zone at E19.5 compared to vehicle treatment. (**A**) Percentage of αSMA+ pericytes area and representative IHC for aSMA staining in labyrinth zone. (**B**) Percentage of collagen IV staining and representative IHC for collagen IV staining in the labyrinth. (**C**) Percentage of laminin staining and representative IHC for laminin staining in the labyrinth. (**D**) Percentage of PAS staining and representative PAS images in the labyrinth. 6-images/placenta were taken at 40x, graphs represent mean ± SEM, Significance; Student’s t-test (*P < 0.05, ***P < 0.001). Scale bars in (**A–C**) = 120uM; in (**D**) = 30 uM.

With fetal blood space altered, components that contribute to blood space formation, structure, integrity and function were further evaluated. It is well established that pericytes associate with endothelial cells and wrap around the walls of the fetal capillaries in the placenta. In addition to providing structural support, they, along with trophoblast and fetal endothelial cells contribute to the extracellular matrix (**ECM**) of the placenta and are suggested to play a role in vascular remodeling and maturation^66^. Immunohistochemistry (**IHC**) revealed that α-SMA^+^ labyrinth pericyte area was increased (p < 0.05; Fig. 5A) in the placentae from Δ9-THC exposed dams, when compared with vehicle treated controls. Collagen IV, an ECM component, was increased (p < 0.05; Fig. 5B), while laminin, another ECM component, was not significantly altered (Fig. 5C). Notably, there was increased PAS staining in the labyrinth zone of Δ9-THC placentae (p < 0.01) but given that diastase treatment did not affect this PAS staining, this is not attributed to an increase in glycogen storage (Fig. 5D). Likely, the increased PAS staining was reflective of changes to components of the ECM/basement membrane.

### Δ9-THC exposure results in reduced GLUT1 and GR *in vivo*

**Figure 6:**
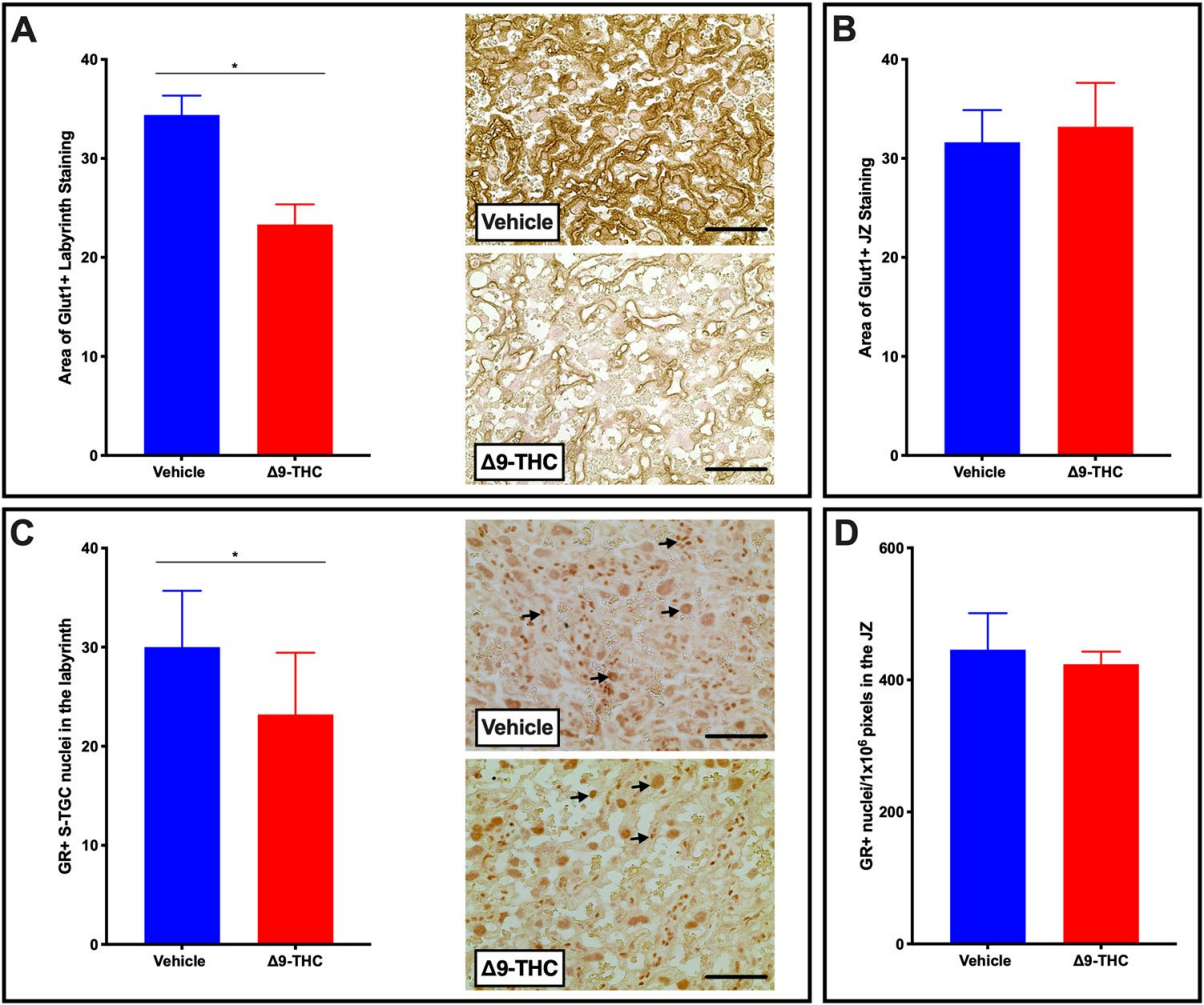
Exposure to 3 mg/kg Δ9-THC during gestation leads to decreased GLUT1 and GR exclusively in the labyrinth zone at E19.5 compared to vehicle treatment. (**A**) Percentage of GLUT1 area and representative IHC for GLUT1 in the labyrinth layer of placentae from vehicle and Δ9-THC exposed dams. (**B**) Percentage of GLUT1 area junctional zone. (**C**) Percentage of GR area and representative IHC for GR in the labyrinth layer of placentae from vehicle and Δ9-THC exposed dams. (**D**) Percentage of GR area in the junctional zone. For labyrinth layer, 6-images/placenta were taken at 40x, while for junction zone 6-images/placenta were taken at 10x. Graphs present mean ± SEM, Significance; Student’s t-test (*P < 0.05). Scale bars = 30 uM. Arrows indicate positive staining for GR in (**C**).

The placenta adapts its nutrient transport system in response to the maternal environment. Glucose is the primary nutrient required for the growth of both the placenta and the fetus. The fetus is dependent on glucose uptake from maternal circulation across the interhemal membrane of the placenta by members of the facilitated glucose transporter family (GLUTs). *GLUT1* is the primary glucose transporter and is highly expressed in the placenta throughout both rodent and human pregnancy^45,67–69^. As the primary glucose transporter, GLUT1 is regularly evaluated in several models of IUGR^64,68,70–72^. Thus, upon observation of fetal growth restriction and altered placental blood spaces in placentae from Δ9-THC pregnancies, the expression of GLUT1 was evaluated. GLUT1 was not altered in the junctional zone of placentae from Δ9-THC exposed dams; however, it was significantly reduced in the labyrinth layer (p < 0.05; Fig. 6A,B). A transgenic glucocorticoid receptor deficient mouse study has previously demonstrated that reduced placental GR expression is accompanied by a decrease in GLUT1, resulting in growth restricted pups^73^. As Δ9-THC has been shown to interact with glucocorticoid receptor (**GR**)^74,75^, and GR-signaling mediates GLUT1 expression^73,76^, GR expression was evaluated in both placental zones. Interestingly, GR positive nuclei were reduced in the labyrinth layer of Δ9-THC placentae (p < 0.05), but not the junctional zone (Fig. 6C,D).

### Δ9-THC exposure in human trophoblast results in reduced GLUT1 and GR, *in vitro*

**Figure 7:**
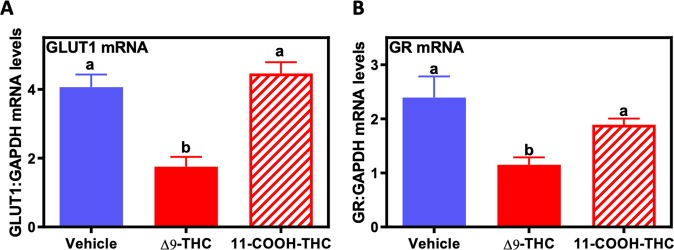
Δ9-THC decreases *GLUT1* and *GR* in human BeWo trophoblast cells. Real-time qPCR of human BeWo cells treated with either vehicle, 15 µM Δ9-THC, or 15 µM 11-COOH-THC for 24 hours. Total RNA was extracted and reverse-transcribed to cDNA and normalized to *GAPDH*. All values were expressed as mean ± SEM (N = 6/group). Significant differences between treatment groups determined by 1-way ANOVA. Different letters represent means that are significantly different from one another according to Tukey’s post test (*P < 0.05, **P < 0.001).

It is of paramount importance, when using animal models to study human pregnancy related pathology, to evaluate whether observations are of relevance to the human. BeWo cells were derived from a human choriocarcinoma and are well published as a model of human villous trophoblast^77–79^, and have been used as an *in vitro* model to examine the effects of Δ9-THC on placental function^12,80–82^. Thus BeWo cells were cultured with and without 15 µM Δ9-THC or its inactive metabolite, 11-COOH-THC, to explore the direct effects of Δ9-THC on *GLUT1* expression. 15 µM was chosen as the experimental dose based on studies, which determined equivalent doses to those found in the serum of *cannabis* users and did not affect cellular viability in BeWo cells^12,80,82,83^. Treatment with Δ9-THC led to decreases in the steady-state mRNA levels of *GLUT1* and *GR* (p < 0.05), while the metabolite (11-COOH-THC) at an equimolar concentration, had no effect (Fig. 7A,B).

## Discussion

Epidemiological studies link perinatal *cannabis* use with low birth weight outcomes, though little is known about whether Δ9-THC alone underlies the fetal growth restriction observed^6–10^. While this is not the first study to show that 3 mg/kg Δ9-THC causes fetal growth restriction, we do believe that it is the first study in rats to demonstrate that this dose leads to symmetrical IUGR with post-natal catch up without any compromise to maternal outcomes. This is of significance as IUGR with post-natal catch up is a strong predictor of long-term metabolic disease^53,84^, thus, this may explain why Δ9-THC rat offspring exhibit long-term glucose intolerance^85^ and adverse neurobehavioural outcomes^47,86–88^. Further, building on the mouse study showing that at 5 mg/kg *i.p*. Δ9-THC placental pathology included both the junctional zone and the labyrinth layer, the current study in rat demonstrates that a lower dose of 3 mg/kg *i.p*. induces only a labyrinth-specific alteration in maternal and fetal blood space with decreased labyrinth expression of the glucose transporter, GLUT1. Collectively, we believe that this model in the rat may prove useful for additional metabolic and placenta studies, as there is no fetal demise.

Increased placental weight has been observed in *cannabis* users and in mice exposed to *cannabis* smoke during pregnancy^53,89^, therefore it is possible that it is the Δ9-THC in *cannabis* that contributes to this reported increase, though it is worth noting that 5 mg/kg Δ9-THC *i.p*. led to decreased placental weights in the mouse^12^. This could be attributed to the noted loss of junctional zone trophoblast subtypes at that dose and may identify a population of trophoblast that are more susceptible to Δ9-THC. As the body of Δ9-THC and *cannabis* research gets larger, it will be important to recognize that dose, delivery method and species may contribute to differential results between studies.

Like other models of placental insufficiency that identify changes in the relative size of placental layers^64,65,90^, this study identifies that placentae from pregnancies exposed to Δ9-THC exhibited changes in the labyrinth layer, but not in the junctional zone. While proliferation at E19.5 was not the cause for the increased area of the labyrinth layer from these pregnancies, there is the possibility that there was proliferation of trophoblast and endothelial cells at an earlier time in gestation, which may have contributed to the altered size. The increased area of the fetal blood space-associated α-SMA^+^ pericytes and the maternal blood space of the labyrinth may contribute to the larger labyrinth size in the placentae from Δ9-THC exposed dams. Those factors contributing to the labyrinth size likely also contribute to the increased placental weight. Further contributing to the heavier placental weight, it remains possible that the decidual zone was also larger (not assessed in the current study).

The decreased fetal blood space and increased maternal blood space created a 60% higher maternal to fetal blood space ratio in the placentae from Δ9-THC exposed pregnancies, suggestive of impaired nutrient transport^91^. The changes in the ratio of maternal to fetal vascularity could be attributed to an overall defect in blood vessel formation given pregnant women who used *cannabis* at least once per month exhibited less expression of CD31 in the placenta, a marker of endothelial cells and thereby, indirectly, angiogenesis^12,91^. Moreover, treatment of pregnant mouse dams with 5 mg/kg *i.p*. also demonstrated narrow blood vessels and lower CD31 expression in the placenta, suggesting Δ9-THC may impair blood vessel formation^18^. Our current study identifies compromised blood vessels at only 3 mg/kg *i.p*. and revealed pericytes and collagen deposition as potential contributors. The fetal blood space, while reduced in area exhibit increased fetal blood space perimeter/area ratio. Pericytes stabilize the endothelial lined fetal vasculature as they deposit basement membrane matrix^66^. Thus, it is noteworthy that the area of both pericytes and Collagen IV staining in the labyrinth of Δ9-THC exposed pregnancies was increased. Whether this implies that the vasculature in these placentae is hyper mature is not known. It is important to consider that the increased pericytes and collagen may contribute to the reduced fetal blood space observed. However, the underlying mechanisms promoting disproportionately higher maternal to fetal blood area in the labyrinth zone are elusive. It is tempting to speculate that the higher ratio in the placentae from Δ9-THC exposed dams could be a reflection of a lack of development of extensive branching of the fetal capillary network into the maternal blood spaces. As a result, the maternal blood spaces would appear smaller in vehicle controls where normal branching had occurred when compared to placentae from Δ9-THC exposed dams. This could be a result of aberrant signaling between trophoblast, pericytes and endothelial cells in the labyrinth. Angiogenic signals, including Pdgfb and Vegfa are produced by these cells and are essential for development of the fetal capillary network in the labyrinth^92–94^. The specific roles of each cell type and how they interact is not well understood; however, it is likely to be important in understanding phenotypes like the one observed in this study.

Placental glucose transport is critical for proper fetal development and elegant studies in the human placenta have demonstrated that glucose transporter proteins (including GLUT1) facilitate a net glucose transfer from maternal circulation to the fetus^95,96^. In human pregnancy, *GLUT1* is the primary placental glucose transporter, while both *Glut1* and *Glut3* mediate glucose transport in the late gestation rodent placenta. Given its localization at the site of maternal-fetal exchange in both the rodent and human, it is not surprising that fetal over-growth is associated with higher placental GLUT1 expression, while lower expression is linked to fetal growth restriction^68,97–99^. Our studies revealed that exposure to Δ9-THC during gestation led to ~35% lower placental GLUT1 expression in the labyrinth layer of the E19.5 rat placenta. The decrease in GLUT1 at this time point was concomitant with a decrease in the fetal to placental weight ratio but preceded the symmetrical growth restriction observed at parturition. Other models of placental insufficiency-induced fetal growth restriction have observed similar decrease in labyrinth expression of GLUT1^64^. Previous *in vivo* studies have reported that Δ9-THC and other cannabinoids can alter glucose transport in the brain and adipose, however, to our knowledge, this study is the first to report a decrease in the placental glucose transporter, GLUT1^100,101^. Further, we demonstrated that GR, which is critical for the expression of placental GLUT1, was decreased specifically in the labyrinth layer of the Δ9-THC rat placenta^73–75^. Importantly, acute glucocorticoid elevation results in increased GR expression, however prolonged exposure leads to decreased GR (reviewed in^102^). As Δ9-THC is reported to increase circulating cortisol/corticosterone levels^103–106^, we speculate that chronic maternal exposure to Δ9-THC may lead to increased maternal glucocorticoid release and ultimately decreased GR and GLUT1 expression. Future studies are warranted in trophoblast cells to further implicate this direct relationship. Alternatively, Δ9-THC has also been shown to bind the glucocorticoid receptor and therefore, may also act to cause a decrease in GR expression over time^75^. This theory is supported by our findings in human BeWo cells, in which Δ9-THC had *direct* effects to decrease steady-state levels of both *GR* and *GLUT1* mRNA, whereas its metabolite, THC-COOH did not. Therefore, the Δ9-THC-induced decrease in *GLUT1* may underlie the previously observed effects of Δ9-THC to decrease glucose transport in BeWo cells^107^. Further studies are warranted to examine whether GR and GLUT1 expression is impaired in other fetal and neonatal organs. To confirm the functional role of diminished placental GLUT1 with Δ9-THC-induced fetal growth restriction, rescue experiments with over-expression of placental GLUT1 will be required to determine whether placental insufficiency and adverse neonatal outcomes could be reversed.

Based on the results in this study, the reduction in fetal growth are likely attributable to impaired placental function, however, as Δ9-THC has been shown to cross the placenta, it is worth considering that Δ9-THC binding to the CB1R/CB2R in the fetal liver and brain may also have an impact^12,82,100,108^. The current study is limited in its scope and independent studies will need to be conducted to evaluate the post-natal onset of MetS along with an in-depth evaluation of the placenta vascular pathology. The placenta assessment did not examine the timing of the onset of Δ9-THC, or the effect of Δ9-THC on interhemal membrane thickness, endothelial population, nor the expression of the cannabinoid receptors. An assessment of each of these factors, while beyond the scope of this study would significantly contribute to our global understanding of the effect of Δ9-THC on the placenta.

In summary, while clinical studies examining *cannabis* use in pregnancy on placental outcomes are confounded by socioeconomic status and other drug use, we have demonstrated that Δ9-THC alone during pregnancy can lead to placental insufficiency resulting in symmetric fetal growth restriction. Importantly, this can occur without alterations to fetal viability, litter size, or maternal weight gain. Moreover, we have identified that defects in fetal blood space area and GLUT1 expression specifically in the labyrinth zone, the site of maternal-fetal exchange, likely underlies these defects in placental function. Given the strong links between placental-insufficiency, induced fetal growth restriction and metabolic disease risk, there is a great impetus to examine the short and long-term effects of gestational Δ9-THC exposure on the fetus/placenta and the affected offspring, respectively^84^. This is especially urgent considering the greater legal access to *cannabis*, rising Δ9-THC concentrations, and the perception by pregnant women that *cannabis* use poses no risk to the fetus^109,110^. As such, targeting the education of *cannabis* use during pregnancy among young, urban, socioeconomically disadvantaged women will be critical.

## Materials and Methods

### Animals and experimental paradigm

All procedures were performed according to guidelines set by the Canadian Council on Animal Care with approval from the Animal Care Committee at The University of Western Ontario. Pregnant female Wistar rats (250 g) were purchased from Charles River (La Salle, St. Constant QC), shipped at E3, and left to acclimatize to environmental conditions of the animal care facility for three days. For the entire experimental procedure, dams and offspring were maintained under controlled lighting (12:12 L:D) and temperature (22 °C) with *ad libitum* access to food and water^82^. Dams were randomly assigned to receive a daily dose of vehicle (1:18 cremophor: saline *i.p*.) or Δ9-THC (3 mg/kg *i.p*) from E6.5 to E22 (N = 14 total, N = 8 dams/group which delivered and N = 6 dams/group for E19.5 analysis). This dose and route of injection has been safely used during rat pregnancy in several studies^12,17,19,25^ and has also been demonstrated to not to alter maternal bodyweight, male-female ratio, or litter size^19^. Δ9-THC treatment was initiated at E6.5 in this design since administration of the drug at earlier stages of pregnancy can induce spontaneous abortions^111^.

Maternal body weight and food consumption were monitored daily for the duration of the study to assess pregnancy weight gain, as previously described^112^. Dams were allowed to deliver normally. At birth (postnatal day 1; PND1), pups were weighed and sexed, and litters were culled to 8, preferentially selecting 4 male and 4 female offspring, to ensure uniformity of litter size between treated and control litters. For each dam, gestation length, litter size, birth weight and the number of stillbirths were recorded. From these data the live birth index ([# of live offspring/# of offspring delivered]*100), and the proportion of pups, which were small for gestational age were also determined. At birth, liver and brain weights for culled pups were measured to calculate liver to bodyweight and brain to bodyweight ratios as an assessment of growth restriction. The remaining pups were used to calculate the percent survival to PND4 (as an indicator of neonatal health) and were sacrificed at 3 weeks to determine liver to bodyweight and brain to bodyweight ratios as indicators of postnatal catch-up growth.

At E19.5, a cohort of dams (N = 6 per group) was sacrificed for the determination of litter size, placental weight, fetal weight, and fetal:placental weight ratio. The number of resorptions/litter was also assessed. Placentae from both experimental cohorts were collected and fixed in 4% paraformaldehyde, followed by processing, embedding in paraffin and sectioning for histochemical analysis. In addition, placentae from both experimental cohorts were flash frozen in liquid nitrogen for RNA analysis.

### Immunohistochemistry

All histology was performed on 7 μm sections. All assessments (unless otherwise indicated) were performed on randomly selected slides from a minimum of 3 placentae per treatment group. Semi-quantitative assessment was performed blinded and repeated by a second person. Images were taken using an EVOS XL microscope (Life Technologies, USA). Staining was semi-quantitively assessed by measuring the area of positive stain as a fraction of the total tissue area as previously described^64,90^, unless otherwise specified.

### Labyrinth and junctional zone size

Iso-Lectin B4 staining was performed as previously published^64,90^ and visualized as per manufacturer’s protocol, using DAB (DAKO, USA). Images were taken at low (4x objective) magnification and merged using Photoshop™ to show the entire placenta. Iso-Lectin B4 binds basement membrane under the fetal endothelial cells that line the fetal blood space; thus, positive staining highlights the labyrinth zone. Using Image J, manual measurement of the areas of the labyrinth layer (as defined by the Iso-Lectin staining) and the junctional zone (as defined by parietal trophoblast giant cells, differentiating junctional zone from maternal decidua) was calculated^64,65,90^. The sizes of the junctional and labyrinth zones are reported as the percentage of the total placenta area.

### Placental composition

As a measure of junctional zone composition, glycogen and spongiotrophoblast assessment was performed using hematoxylin and eosin (H&E) stained images (10x objective). For each placenta, the respective areas of glycogen trophoblast and spongiotrophoblast were presented relative to junctional zone area. Within the labyrinth, as an assessment of placental vasculature, slides stained for Iso-Lectin B4 were used to assess maternal and fetal blood space with 6-images/placentae taken at high magnification (40x objective). All Iso-Lectin-positive blood spaces were identified as fetal capillaries, which are referred to as fetal blood space, while all Iso-Lectin negative blood spaces with an associated S-TGC (as identified by their large nuclei) were identified as maternal blood space; area and perimeter of both fetal and maternal blood space were measured using Photoshop/Image J, with area represented as a percentage of each field of view. Supporting the fetal capillaries are the pericytes, identified by αSMA and extracellular matrix components, including collagen and laminin. αSMA (ab5694; 1:200), Collagen IV (Abcam ab6585; 1:200) and Laminin (Abcam 11575; 1:200) immunohistochemical staining in the labyrinth was assessed using images (10x objective) with the area of positive staining presented as a percentage of the field of view. Sinusoidal Trophoblast Giant cells (S-TGC) line the maternal blood spaces. Thus, the area of S-TGC nuclei was measured and all positive nuclei with an area equal to or larger than the smallest S-TGC nuclei were counted as a positive S-TGC. The same technique was used to assess number of Ki67 (Abcam 16667; 1:100) positive nuclei, as a measure of proliferation. GLUT1 (Abcam ab652; 1:300) and, GR (Proteintech 24050-1-AP; 1:200) immunohistochemical staining was performed with labyrinth and junctional zone assessment. Junctional zone assessment used images (10x objective), with the number of positive nuclei counted for each GR image and area of positive stain measured for each GLUT1 image. Labyrinth assessment was performed in the same manner, however, 6 images/placenta were used (40x objective) so that clustered nuclei were not miscounted, as positive nuclei in the labyrinth had much closer proximity to one another than in the junctional zone. While PAS staining is used as a measure of glycogen and/or aldohexose content in the glycogen trophoblast of the junctional zone, in the labyrinth it more commonly identifies extracellular matrix. Staining was performed as per manufacturer’s protocol (Sigma, USA), both with and without diastase treatment on serial sections. 6-images/ placenta were taken (40x objective) in both the labyrinth and junctional zone, and positive staining was measured and presented as a percentage of area of field of view.

### Cell Culture and Δ9-THC treatment

To confirm that the effects of Δ9-THC on placental GLUT1 and GR expression were a direct effect, we tested the effects of Δ9-THC exposure on human BeWo cells *in vitro*. The BeWo cells have been widely used as an *in vitro* model for drug (*i.e*. Δ9-THC) studies^12,80,113^. As previously described^82^, cells (passages 8–18) were cultured in 75-cm^2^ flasks in F-12K Nutrient medium (Gibco) with 10% fetal bovine serum (Gibco) and 1% Penicillin/Streptomycin at 37 °C in 5% CO_2_ in air.

To test the effect of Δ9-THC on GLUT1 and GR expression, BeWo cells were plated on a 12-well plate with 2 × 10^5^ cells per well in 1 mL of F-12K Nutrient medium and allowed to attach for 24 hours as previously described^82^. Briefly, following the 24-hour incubation period, media was removed and replaced with treatment media containing 15 μM of Δ9-THC (dissolved in final concentration of 0.1%(v/v) ethanol, Cayman Chemicals, Ann Arbor, MI) or 0.1%(v/v) ethanol (vehicle control). The 15 µM dose of Δ9-THC was chosen based on previous pharmacokinetic studies which determined equivalent doses to those found in the serum of *cannabis* users^80,83^. Additionally, BeWo cells were treated with 15 μM 11-COOH-THC (Sigma-Aldrich), the main metabolite of THC, to assess its potential effects on GLUT1 and GR expression. The 24-hour time-point allowed for detection of changes in the steady-state mRNA levels of placental target genes, as previously published^82^.

### RNA isolation and real-time PCR analysis

As we have previously published^82^, total RNA was extracted from frozen E19.5 placenta and BeWo cells using TRIzol reagent (Invitrogen) and chloroform (Sigma-Aldrich) in a standard TRIzol/chloroform extraction protocol as described by the manufacturer. Following precipitation, total RNA was collected from the pellet and dissolved in DEPC-treated water. Deoxyribonuclease I, Amplification Grade (Invitrogen) was added to the RNA to digest contaminating single- and double-stranded DNA. Four micrograms of RNA were reverse-transcribed to cDNA using random hexamers and Superscript II Reverse Transcriptase (Invitrogen). Primer sets directed against human *GLUT1* (Forward; 5′-GGACCTTCGATGAGATCGCT-3′ and Reverse; 5′-TCTTGTCACTTTGGCTGGCT-3′) and *GR* (Forward; 5′-GGACCACCTCCCAAACTCTG-3′ and Reverse; 5′-GCTGTCCTTCCACTGCTCTT-3) gene targets of interest were designed through National Center for Biotechnology Information’s primer designing tool and generated via Invitrogen Custom DNA Oligos. For rat placental real-time PCR analysis, primer sets were targeted against *Epcam1* (Forward; 5′-CGCAGCTCAGGAAGAATGTG-3′ and Reverse; 5′-TGAAGTACACTGGCATTGACG-3′) and *Gcm1* (Forward; 5′-CCCCAACAGGTTCCACTAGA-3′ and Reverse; 5′-AGGGGAGTGGTACGTGACAG-3′). Quantitative analysis of mRNA expression was performed via RT-PCR using fluorescent nucleic acid dye SsoFast EvaGreen supermix (BioRad) and BioRad CFX384 Real Time System. The cycling conditions were 95 °C for 10 min, followed by 43 cycles of 95 °C for 15 sec and 60 °C for 30 sec and 72 °C for 30 sec. The cycle threshold was set so that exponential increases in amplification were approximately level between all samples. Relative fold changes were calculated using the comparative cycle times (Ct) method, normalizing all values to the geometric mean of the housekeeping gene, human *GAPDH* (Forward; 5′-AGGTCCACCACTGACACGTT-3′ and Reverse 5′GCCTCAAGATCATCAGCAAT-3′) or rat *Gapdh* (Forward; 5′-TAAAGAACAGGCTCTTAGCACA-3′ and 5′-AGTCTTGGAAATGGATTGTCTC-3). *GAPDH* was determined as a suitable housekeeping gene using algorithms from GeNorm, Normfinder, BestKeeper, and the comparative ΔCt method to place it as the most stable housekeeping gene from those tested (*e.g*. β-actin, 18 S ribosomal RNA)^114–117^. Given all primer sets had equal priming efficiency, the ΔCt values for each primer set were calibrated to the average of all control Ct values, and the relative abundance of each primer set compared with calibrator was determined by the formula 2^ΔΔCt^, in which ΔΔCt was the normalized value^82^.

### Statistical analyses

All statistical analyses were performed using GraphPad Prism 8 software. Results were expressed as means of normalized values ± SEM, and the threshold for statistical significance was set as *P* < 0.05. A sample size of 7–8 offspring (*i.e*., litter is the statistical unit) was used for all *in vivo* experiments, as this provided enough statistical power to detect significant differences in outcome measures. All cell culture experiments were performed in biological replicates of 3, where each replicate represents an independent experiment using a different frozen cell stock or passage number. For all maternal, fetal, and neonatal outcomes, IHC, and real-time PCR, Student’s unpaired t-tests were performed to assess significance (P < 0.05).

## Supplementary information

Supplementary Information.
