# Supplementary material for: Δ9-tetrahydrocannabinol exposure during rat pregnancy leads to symmetrical fetal growth restriction and labyrinth-specific vascular defects in the placenta
Source: Sci Rep. 2020 Jan 17;10:544. doi: 10.1038/s41598-019-57318-6 (PMC6969028; doi:10.1038/s41598-019-57318-6)
Supplement: Supplementary file 1 — Supplementary Information. [file 41598_2019_57318_MOESM1_ESM.pdf]

Δ9-tetrahydrocannabinol exposure during rat pregnancy leads to symmetrical fetal growth restriction and labyrinth-specific vascular defects in the placenta.

Bryony V. Natale[1,2], Katarina N. Gustin[2], Kendrick Lee[9,10], Alison C. Holloway[4], Steven R. Laviolette[5,10], David R.C. Natale**[1,2,3], and Daniel B. Hardy*[6-10]

[1]Department of Obstetrics and Gynaecology, Queen's University, Kingston, Canada.
[2]Department of Obstetrics, Gynecology & Reproductive Sciences, School of Medicine, University of California San Diego, La Jolla, CA, USA
[3]Department of Biomedical and Molecular Sciences, Queen's University, Kingston, Canada.
[4]Department of Obstetrics and Gynecology, McMaster University, Hamilton, Canada
[5]Department of Anatomy and Cell Biology, [6]The Children's Health Research Institute, [7]Lawson Health Research Institute, The Departments of [8]Obstetrics and Gynaecology and [9]Physiology and Pharmacology, [10]The University of Western Ontario, London, Ontario, Canada, N6A 5C1

Supplemental Figure and Tables:

Supplemental Table 1. Forward and reverse sequences for the rat primers used for quantitative Real-Time PCR.

Gene	*Forward*	*Reverse*	*GenBank/ Reference*
Gcm1	CCCCAACAGGTTCCACTAGA	AGGGGAGTGGTACGTGACAG	NM_017186
Gapdh	CAAGTTCAACGGCACAGTCA	CCCCATTTGATGTTAGCGGG	NM_017008.4

Supplemental Figure 1. Exposure to 3 mg/kg Δ9-THC during gestation has no effect on expression of *Gcm1* mRNA in E19.5 placenta. Real-time qPCR of rat placentae treated with vehicle or Δ9-THC throughout gestation beginning at E6.5. Total RNA was extracted and reverse-transcribed to cDNA and normalized to *Gapdh*. All values were expressed as mean±SEM (N=6/group).

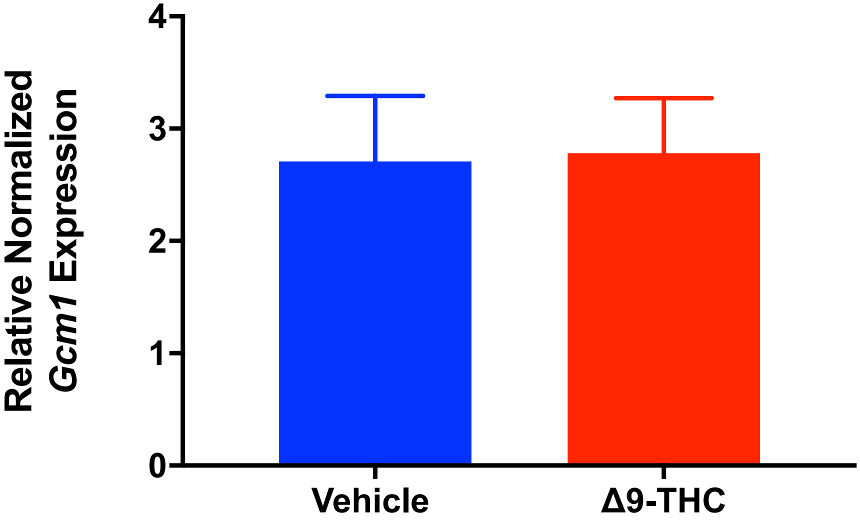

Relative Normalized
Gcm1 Expression
4
3
2
1
0
Vehicle
Δ9-THC
